# Conventional Gel Electrophoresis-Resolvable Insertion/Deletion Markers for Individual Identification and Analysis of Population Genetics in Red-Crowned Cranes in Eastern Hokkaido, Japan

**DOI:** 10.3390/ani12172293

**Published:** 2022-09-04

**Authors:** Erika Kawasaki, Dong Wenjing, Akira Sawada, Momoko Nakajima, Kunikazu Momose, Tomoo Yoshino, Tomoko Amano, Daiji Endoh, Nobuyoshi Nakajima, Hiroki Teraoka

**Affiliations:** 1 School of Veterinary Medicine, Rakuno Gakuen University, Ebetsu 069-8501, Japan; 2 Biodiversity Division, National Institute for Environmental Studies, Tsukuba 305-8506, Japan; 3 NPO Red-Crowned Crane Conservancy, Kushiro 085-0036, Japan; 4 Kushiro Zoo, Kushiro 085–0201, Japan; 5 College of Agriculture, Food and Environment Sciences, Rakuno Gakuen University, Ebetsu 069-8501, Japan

**Keywords:** InDel, individual identification, *Grus japonensis*, Japan, HTS

## Abstract

**Simple Summary:**

The red-crowned crane is an endangered bird species in the Far East Eurasian continent and Hokkaido, Japan. Individual identification has been achieved with banding and installed transmitters in only a few cranes, though individual identification is essential for various assessments of cranes on their behavioral characteristics, for example. An insertion/deletion (InDel) mutation is a mutation ranging from 1 to 50 bp and is very useful for genetic studies. If primer sets across InDels (>20 bp) can be designed, an InDel polymorphism can be determined with conventional agarose gel electrophoresis, as reported in some plant species. We found a sufficient number of InDel primer sets to be used for individual identification and analysis of population genetics of red-crowned cranes in Hokkaido. The method has additional advantages, such as convenience and low cost, without sequencing and an expensive apparatus.

**Abstract:**

Red-crowned crane *Grus japonensis* is an endangered species in two separate populations: the mainland population in the Eurasian continent and the island population in eastern Hokkaido, Japan. We found 11 insertion/deletion (InDel) markers in the genome of the red-crowned crane and designed primer sets across these InDels that can be analyzed with conventional agarose gel electrophoresis. Sixty-six samples of whole blood and skeletal muscle obtained from red-crowned cranes, including 12 families in eastern Hokkaido from 1994 to 2021, showed different patterns in gel images of 11 InDel PCR reactions except for two pairs. The combined non-exclusion probability of the 11 markers indicates that individuals can be determined with a probability of 99.9%. In 39 non-relative chicks, the expected heterozygosity (He) was 0.316, suggesting low genetic diversity. This might not be caused by high levels of inbreeding since the average FIS was not significantly different from zero (0.095, *p* = 0.075). The results suggest that the 11 InDel primer sets can be used for fairly accurate individual identification as well as genetic population analyses in red-crowned cranes in the island population.

## 1. Introduction

The red-crowned crane *grus japonensis* is an endangered bird species (IUCN Red List, https://www.iucnredlist.org/es/species/22692167/93339099) (accessed on 1 April 2022) in two separate populations: the mainland population in the Far East Eurasian continent and the island population in Japan. The total population of red-crowned cranes in the world was estimated to be 4070 in 2021 [1]. Red-crowned cranes in the mainland population raise their siblings in the Amur River Basin in summer and migrate to the Korean Demilitarized Zone (DMZ) between North Korea and South Korea as well as the east coast of middle China for wintering [2]. In contrast, red-crowned cranes in the island population live in their territories in summer and spend winter in some major feeding stations in which corn is supplied by the Ministry of Environment, Japan (MOEJ) and minor private stations within eastern Hokkaido as a resident population [3].

Since mitochondria in sperm are removed away from the oocyte just after fertilization, the mitochondrial genome is maternally inherited, resulting in high conservancy without genetic recombination [4]. The d-Loop, a non-coding region in the mitochondrial genome, showed a very high frequency of mutation, 5–10-times higher than that in somatic DNA, and it has been studied for genetic analyses such as analysis of genetic diversity within a species [5], including some crane species [6,7,8]. Haplotype analyses clearly suggested that the genetic diversity of red-crowned cranes in the island population is very poor, and only three haplotypes (Gj1, Gj2 and Gj3) were confirmed, whereas 14 haplotypes (Gj4-Gj6/18) were confirmed in the continental population [9,10,11]. It is speculated that this scarce diversity of red-crowned cranes in the island population might be caused by bottleneck effects since they were nearly extinct at the end of the 19th century [12,13].

The number of red-crowned cranes in the island population can be estimated relatively easily now by direct counting since most of the cranes gather in some major feeding stations in eastern Hokkaido in winter. However, individual identification is necessary for estimating life expectancy and for studying various aspects of social behavior, such as the mechanism for pairing or the territorial system. The greatest fear in terms of crane conservation at present is the outbreak of a deadly and highly infectious disease. In this context, individual identification can also contribute to disease control.

In bird species, banding has been very useful for obtaining information on reproductive biology and social structures, including the territorial system [14]. A red-crowned crane chick in Bettouga in Nemuro City in eastern Hokkaido, Japan, was banded for the first time by a group at the Yamashina Institute for Ornithology in 1988. Since the NPO red-crowned crane conservancy (RCC) took over the banding project in 2005, 20–30 chicks have been banded annually, and blood has been collected. More than 400 chicks had been banded by the summer of 2021 (http://www6.marimo.or.jp/tancho1213/hyousikityou202112.pdf) (accessed on 1 April 2022). However, only 1.5–2-month-old flightless chicks with grown legs similar to an adult can be banded after consistently checking their growth in all areas in eastern Hokkaido, Abashiri, northern Hokkaido and also the central Hokkaido area. Ten to twenty volunteers are needed for each banding in order to prevent a chick from fleeing and hiding in a bush. Furthermore, the volunteers sometimes cannot go near wetlands due to the risk of sinking. 

A genetic analysis-based method can be used for individual identification. Red-crowned cranes found dead in fields in eastern Hokkaido have been kept in a freezer since the end of the 1980s, entrusted by MOEJ. Whole blood has been collected from chicks during banding by RCC. Feathers and feces found in the field are also valuable samples for genetic analysis because they contain the genomic DNA of the host birds [15]. The use of simple sequence repeat (SSR) markers is one of the representative methods [16]. SSR markers have been reported for red-crowned cranes in both the island population [17,18] and continental population [19]. While an SSR is a traditional marker for individual identification, it should be analyzed with a capillary sequencer after finding SSR markers and primers to detect. Analysis with an SSR marker also needs relatively intact genomic DNA. Single nucleotide polymorphisms (SNPs) have been one of the most popular markers in genome-wide association studies; however, DNA sequencing is needed to detect SNPs [20]. Although a few SNPs in the major histocompatibility complex (MHC) have been found in red-crowned cranes in the island population, they are not sufficient for the identification of individual cranes [21]. 

An insertion/deletion (InDel) mutation is a mutation ranging from 1 to 50 bp [22]. Since a relatively long InDel (>20 bp) rarely returns to the original wild type [23], InDel markers are very useful for genetic studies. If primer sets across InDels (>20 bp) can be designed, an InDel polymorphism can be determined with conventional agarose gel electrophoresis, as reported in some plant species [24]. Detection of InDel markers is especially useful for sun- or rain-damaged samples for wildlife since the method is widely used for individual identification in forensic samples of humans [25].

The purpose of the study was to find a sufficient number of InDel primer sets to be used for individual identification of red-crowned cranes in the island population. We also used InDel primers to study the basic genetics of the island population. The possible use of InDel PCR for the determination of parent-child cranes and siblings is also discussed.

## 2. Materials and Methods

### 2.1. Samples and DNA Extraction

With permission from the Japanese Ministry of the Environment (MOEJ: Tokyo, Japan) (1704261, 1704281, 1806126, 1806141, 1806151, 1906191), blood was collected from 65 chicks in June and July of 2007–2022 during banding. Blood samples were kept in plastic tubes for a few hours until freezing at −20 °C. A breast muscle of an adult crane (No. R568) that was found dead in 2021 in the Tokachi area (a part of Kushiro City since 2005) and was kept in a freezer in Kushiro Zoo, Kushiro, Hokkaido, was obtained. This crane was banded in the Nemuro area in 1994 (Ring number: T73/R568). 

For most of the whole blood samples (n = 77), genomic and mitochondrial DNA (total DNA) was extracted using the method of Bailes et al. [26]. In brief, 500 µL of STM buffer (64 mM sucrose, 20 mM Tris-HCl pH 7.5, 10 mM MgCl_2_, 0.5% Triton X-100) was added to 5 µL of blood and vortexed. After centrifugation at 3000 rpm for 3 min, 500 µL of STM buffer was added to the resulting pellets and vortexed again. After the second centrifugation, the pellets were incubated with 100 µg/mL (final concentration) pronase at 37 °C for 1 h. For preparation of DNA libraries of six blood samples (Table S1), total DNA was extracted from 5 µL of blood using DNeasy Blood and Tissue Kits (Qiagen, Venlo, The Netherlands), according to the manufacturer’s instructions. A DNA sample was also extracted from 100 mg (wet weight) of muscle using an ISOGENOME Kit (Nippon gene, Tokyo, Japan). A few feathers and feces of cranes were also used to determine whether InDel primers work properly with these samples as templates. Total DNA was extracted from several pieces of feather follicles (usually about 25 mg, n = 4) with DNeasy Blood and Tissue Kits (Qiagen) [27]. The extracted DNA samples were stored at −20 °C until use. Total DNAs that were extracted from intestinal contents of three adult cranes and feces of a chick (wet weight) by Kataoka et al. [28] were also used. 

### 2.2. Search for InDels

DNA libraries were prepared from total DNA samples from three male and three female cranes that were selected from areas as different as possible (Table S1) using the NEB Next Ultra DNA Library Prep Kit for Illumina (New England Bio Labs, Ipswich, MA, USA). Whole-genome sequencing was carried out with HiSeq X Ten and sequence information of at least 20 GB for each crane. Sequence results of 150 bp pair-end reads were analyzed with CLC Genomics Workbench ver. 21.04 (Qiagen). After filtering with a limit of Q20 and 50 bp in length, genome mapping of read sequences obtained was carried out with Mapping Reads to Reference (https://doc.ugene.net/wiki/display/UM/Mapping+Reads+to+Reference) (accessed on 1 March 2021). Registered genome information of red-crowned cranes in the DNA Databank of Japan (DDBJ) (BDFG02000001-BDFG02044166) was used as a reference. More than 1100 different InDels with 30-bp lengths or more were identified using [Local Realignment]-[Basic Variant Detection]. By removing redundant parts around the end of each read and reads with coverage of less than five, 99 Indels consisting of 40 insertions and 59 deletions were selected. The average length of InDels selected was about 32 bp.

### 2.3. Selection of Primer Sets to Detect InDels

In order to detect an InDel mutation by agarose gel electrophoresis, we designed 61 primer sets across the 99 InDels that were selected. PCR reactions were carried out according to the protocol described in the next section. Then, we selected primer sets for which electrophoresis images were obtained as expected. Sequences of positive bands in electrophoresis were confirmed with conventional Sanger sequencing using the ABI PRISM 310 DNA Sequencer (Thermo Fisher, Waltham, MA, USA) for reaction products of the BigDye Terminator v3.1 Cycle Sequencing Kit (Thermo Fisher) [11].

### 2.4. PCR Reactions to Detect InDels

Using our 11 designed InDel primer sets and DNA extracts from blood and a muscle as templates, PCR reactions were carried out with the GoTaq Green Master Mix (Promege, Fitchburg, WI, USA) according to the manufacturer’s instructions with a step down procedure (initial activation of heating at 95 °C for 120 s, 3 cycles of denaturation at 95 °C for 30 s, annealing at 63 °C for 30 s, extension at 72 °C for 30 s, 40 cycles of denaturation at 95 °C for 30 s, annealing at 60 °C for 30 s, extension at 72 °C for 30 s). Electrophoresis with 3% agarose gel was carried out, and patterns of positive bands were visualized with the application of 10 µg/mL ethidium bromide on the gel plate. PCR products were extracted from these positive bands with the FastGene Gel/PCR Extraction Kit (Nippon Genetics, Tokyo, Japan) and used for direct Sanger sequencing for confirmation of the specificity. 

### 2.5. Haplotype Determination and Sexing

The mitochondrial haplotype (control region 2, CR2) [10] was conveniently determined with the amplification refractory mutation system (ARMS) PCR assay using the same DNA extracts from blood samples and a muscle as templates [27]. The ARMS method that can detect Gj1, Gj2, Gj13 and the other haplotypes is almost sufficient because all of the cranes in Hokkaido can be classified into these three haplotypes except very rare individuals of possibly continental origin [11]. Haplotypes of some samples were confirmed by direct Sanger sequencing. Sexing was performed with two sets of primers according to our previous paper [11].

### 2.6. Statistical Analyses

(a) Data sets: Two data sets were used depending on the aim of analyses: (A) genotype data for 39 individuals (“non-kin samples”) and (B) genotype data for 39 individuals (“kin samples”) that included parent and offspring, grandfather and grandchild and twins of 12 families. All non-kin DNA samples are obtained from 39 blood samples. Kin DNA samples were obtained from 38 blood samples and a muscle. Since there are 12 overlaps in non-kin and kin blood samples, total samples for individual identification were 66. Data set A was prepared and used for analyses based on allele frequencies (described in “Basic population genetics”). Because our field procedures force collecting samples based on families, the use of family-based samples for population genetics analysis can bias allele frequency estimates and subsequent results. Therefore, we prepared data set A. Data set B was prepared and used for analysis based on known kin relationships.

(b) Basic population genetics: To describe basic population genetics of the cranes, observed heterozygosity (Ho), expected heterozygosity (He) and polymorphic information content (PIC) were calculated by Cervus [29]. F-statistics (FST: measure of population differentiation, and FIS: measure of inbreeding) were estimated by SPAGeDi [30] based on estimators of Weir and Cockerham [31]. Whether the F-statistics deviated from zero was tested by a permutation test implemented in SPAGeDi. Since FST is just an index, principal component analysis was applied to InDel genotype data to visualize genetic relationships of samples using prcomp function in R. 

(c) Identity analysis: To apply InDel data to individual identification, the data for non-kin samples were analyzed. “Identity Analysis” in Cervus was used to list all pairs that have the same genotypes. A low proportion of such pairs indicates the high ability of the markers as a tool for individual identification. To further evaluate the ability as an individual identification tool, non-exclusion probability of the InDel markers was estimated by “Allele Frequency Analysis” in the program. Non-exclusion probability for identity is the probability of the genotype of two individuals matching by chance (i.e., probability of two samples of different individuals being erroneously judged as the samples of an individual). Since the calculation of the probabilities assumes a Hardy–Weinberg equilibrium (HWE), it was tested by Genepop in advance. Test parameters were: Dememorization = 1000, Batches = 100, Iterations per batch = 1000.

(d) Kin analysis: To apply InDel data to estimation of kin relationships, the data for non-kin samples were analyzed again. “Allele frequency analysis” in the Cervus was used to obtain non-exclusion probabilities for the first parent and parent pair. The former is the probability of the genotype of an individual not being able to be distinguished from possible genotypes of offspring of another individual (i.e., the probability of two samples of different individuals being erroneously judged as a parent and its offspring). The latter is the probability that the genotype of an individual cannot be distinguished from possible genotypes of offspring of the other two individuals (i.e., the probability of three samples of different individuals being erroneously judged as parents and their offspring).

We also considered the application of the concordance rate of genotypes to estimate kin relationships. Here, the concordance rate (%) of InDel genotypes between two individuals was defined as 100 * (number of loci having matched genotypes)/(number of loci). The rate is expected to have a large value if two individuals are kin, whereas a small value is expected if they are non-kin. Therefore, we made two comparisons of concordance rates of kin-samples between known parent-offspring pair vs. non-kin pair and known full-sibling pair vs. non-kin pair. Since the distribution of concordance rates does not necessarily follow a normal distribution, the two comparisons were statistically tested by Wilcoxon’s rank sum test using Excel 2016 with Statcel (the addin forms on Excel-4th ed.) (OMS Publishing, Tokyo, Japan).

## 3. Results

### 3.1. Search for InDel Markers

Whole blood samples from six red-crowned cranes in eastern Hokkaido were used to search for InDel mutations. Sequence data of about 30 G bp were obtained for each crane, corresponding to 20 times the genome size of a red-crowned crane. Using the registered complete sequence of genomic DNA of a red-crowned crane as a reference (https://www.ncbi.nlm.nih.gov/genome/17090) (accessed on 1 March 2021), more than 300 InDel sequences of 30 bp or more were found. After InDels around the end of each contig were discarded, InDel marker candidates that can be used for individual identification were narrowed down to 99.

Across these marker candidates, 61 pairs of primers that produced PCR products of 100–250 bp were designed. These 61 primer pairs were checked with PCR reaction and 3% agarose gel electrophoresis with DNA samples as templates from whole blood samples of six chicks that were used to search for InDels. After these trials, 11 pairs of primers were selected to show expected gel images as calculated in NGS sequencing (Table 1, Figure S1). Gel images of the heterozygous type frequently showed an additional band other than the expected two bands. These extra bands were often found in gel electrophoresis of PCR products for the heterozygous type and were thought to be the result of a heteroduplex mobility shift [32]. PCR products of the 11 InDel primers were confirmed as targets of interest with conventional Sanger sequencing. Other than these, unidentified bands were also found in some cases (Figure S1J).

The 11 InDel primer sets were applied for genomic DNA from four feather shafts of wild cranes found dead in eastern Hokkaido. PCR trials with the 11 InDel primer sets produced the assumed bands in agarose electrophoresis (Figure S2). On the other hand, however, about a half of the PCR products with four InDel primer sets and DNA samples that were extracted from feces of three crane chicks produced positive bands, suggesting that scatological samples are not adequate for our 11 InDel primer sets (Figure S3).

### 3.2. Basic Population Genetics

Using the 11 identified primer sets, InDel genotypes of 39 non-kin samples were determined (Table 2). 

Based on the InDel genotypes of the 39 non-kin samples, observed heterozygosity (Ho), expected heterozygosity (He), F-statistics (FIS), polymorphic information content (PIC), non-exclusion probability for individual identification (NEP) and P-value of test for deviance from the Hardy–Weinberg equilibrium (HWE) (P) were calculated by three regions of eastern Hokkaido (Tokachi, Kushiro and Nemuro) (Table 3 and Table S2). Global He that was calculated together with the 11 primer sets was 0.316, and that of FIS was 0.095. The global FST value obtained in the whole population (39 non-kin samples) was 0.013 with non-significance (*p* = 0.337) (Table 4). 

Pairwise FST values among the three representative regions in eastern Hokkaido are presented in Table 4. FST of Kushiro vs. Nemuro showed a positive value of 0.009 but was not significant (*p* = 0.289). FST of Tokachi vs. Kushiro was −0.006 and was not significant (*p* = 0.566). On the other hand, FST of Tokachi vs. Nemuro showed a value of 0.033441 and approached significance (*p* = 0.050). 

**Figure 1:**
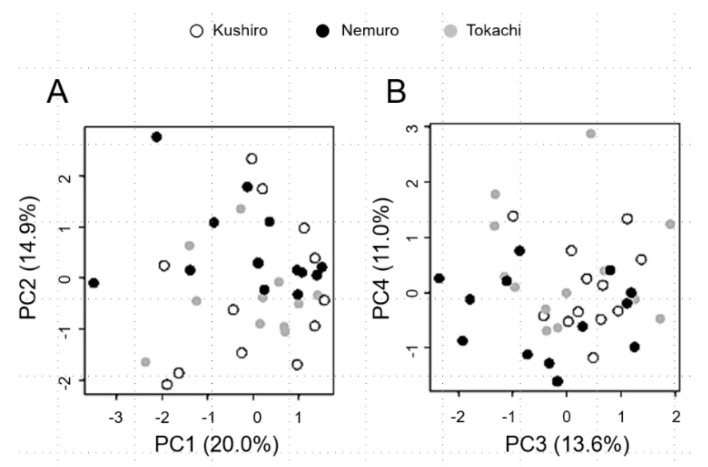
Principal component analysis of InDel genotypes in three populations of Kushiro, Nemuro and Tokachi. (**A**) Biplot of the first and second principal component scores (PC1 and PC2). (**B**) Biplot of the third and fourth principal component scores (PC3 and PC4). Sampling locations are shown by colors: Kushiro (white circles), Nemuro (black circles) and Tokachi (grey circles). Values in parentheses are the contribution ratios of each principal component. Thirteen red-crowned crane chicks were selected from Kushiro, Nemuro and Tokachi in eastern Hokkaido, respectively.

No major difference in principal component scores among groups also indicates non-significant population differentiation (Figure 1). Although Gj1-type cranes are very few (N = 6), the FST value of Gj1 vs. Gj2 was 0.007 (*p* = 0.626) for reference.

### 3.3. Individual Identification

Among the 39 non-kin samples, genotypes of two cranes completely matched (No. 89 vs. No. 127, Table 2). Deviation from the HWE hypothesis was not observed (*p* > 0.05 except Id-03, Table 3 and Table S2). Bonferroni correction ruled out significant deviation in Id-03. Thus, we proceeded with the calculation of non-exclusion probability. Non-exclusion probability of individual identification with the 11 InDel markers was 0.001 (0.1%). This means that individual identification with the markers can be carried out with a probability of 99.9%.

There was another pair of cranes with the same genotype (No. 331 vs. No. 388) in a total of 66 samples, including kin samples that will be mentioned in the next chapter (Table 2 and Table 5). Non-exclusion probability of individual identification with the 11 InDel markers was 0.001 (0.1%). Thus, individual identification in the Hokkaido population with the 11 markers can be carried out with a probability of 99.9%.

### 3.4. Kin Analysis

Based on banding records by the NPO red-crowned crane conservancy, InDel patterns of 39 cranes with known family structures (consisting of parent and child, grandfather and grandchild and twins of 12 families) were investigated (Table 5). 

As the combined non-exclusion probability for the first parent was 0.518 (51.8%), the parent–offspring relationship cannot be determined on the basis of InDel patterns of two cranes. However, the combined non-exclusion probability (parent pair) was 0.077 (7.7%), indicating that the parent–offspring relationship can be estimated with lower accuracy (92.3%) if InDel patterns of couples and another crane for comparison are available. 

**Figure 2:**
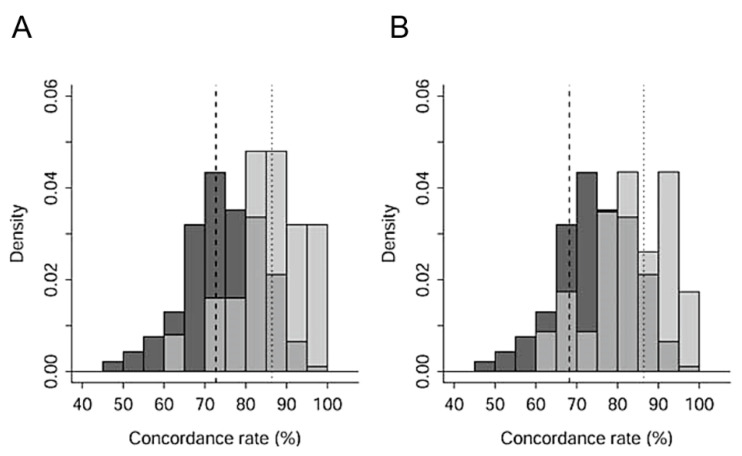
Histograms of concordance rates in pairs of red-crowned cranes in Hokkaido. (**A**) parent–offspring pairs (pale grey) and non-relative pairs (dark grey). (**B**) Full-sibling pairs (pale grey) and non-relative pairs (dark grey). Intermediate grey shows the redundant part of blood relatives and non-relatives in (**A**,**B**). Dotted lines in (**A**,**B**) upper 5% point non-relative pairs. Dashed line in (**A**) lower 5% point of parent–offspring pairs. Dashed line in (**B**) lower 5% point of full-sibling pairs. Parent–sibling pairs, 25; full sibling pairs, 21; non-relative pairs, 366.

Concordance rates of known parent-offspring pairs were significantly higher than those of non-kin pairs (Wilcoxon rank sum test, N1 = 25, N2 = 366, W = 7425, *p* < 0.001). The rates of known full-siblings were significantly higher than those of non-kin pairs (Wilcoxon rank sum test, N1 = 21, N2 = 366, W = 6216, *p* < 0.001). As shown in histograms of these three groups (Figure 2), a pair of cranes with a very high concordance rate was expected to be a parent–child pair or full siblings. The upper 5% point of the distribution of non-relatives was 86.4%, and the lower 5% points of the distributions of parent-offspring and full-siblings were 72.7% and 68.2%, respectively.

Although sample sizes were extremely small, concordance rates of grandfather and grandchild and concordance rates of twins were calculated for reference. Concordance rates of a grandfather and his four grandchildren (N = 4) were 45.5–72.7% (average rate of 56.8 ± 6.6%).

## 4. Discussion

The present study provided primer sets of PCR to detect 11 InDel markers by conventional agarose gel electrophoresis. Two pairs among 66 individual cranes (including some kin cranes) (2 pairs among 2145 possible combinations) matched perfectly. The non-exclusion probability value was 0.1%. Thus, the InDel markers that were found are very useful for individual identification of cranes, with an accuracy of more than 99.9%. PCR with InDel primer sets also produced positive bands in gel electrophoresis with DNA samples from feathers as non-invasive samples other than whole blood and muscles; however, feces were unfortunately not adequate. Detailed information on InDel markers was already reported in domestic chicken (*Gallus gallus*) [33,34].

Banding is undoubtedly useful, but it is very time consuming and needs the cooperation of many people for red-crowned cranes since cranes are able to hide easily in tall reeds, for example, in eastern Hokkaido. In contrast to the immense efforts needed for banding, only one or two days are needed for DNA extraction, PCR and gel electrophoresis for many samples, such as ten or more samples at the same time, if feathers and other samples are available. One of the biggest advantages is that individual identification of samples can be carried out with conventional agarose electrophoresis. It is much cheaper than sequencing. Only a thermal cycler and an apparatus for agarose gel electrophoresis are needed, and these are now inexpensive and are used even in small laboratories such as those in zoos or other facilities.

Microsatellite markers have been used extensively for population genetics and individual identification of bird species, including red-crowned cranes [17,18,19,35], Lear’s macaw (*Anodorhynchus leari*) [36], African grey parrots (*Psittacus erithacus*) [37] and Blakiston’s fish owl (*Bubo blakistoni*) [38]. SNPs have been found in mitochondrial Cyt B of red-crowned cranes in the continental population [35]. Zhan et al. [39] reported 144 SNPs (108 in intron, 36 in exon) in Saker falcon (*Falco cherrug*) in Eurasia, suggesting the importance of functional exonic SNPs for the study of genetics in a widespread avian species. MHC is one of the most functional genes that are related to the immune system with vast diversity [40]. MHC class IIB genes were used for studying population genetics and individual identification in Blakiston’s fish owl of Hokkaido, Japan [41]. Although SNPs in MHS is promising, only a few SNPs in MHC were found in red-crowned cranes as far as we know [21].

SNPs markers were also used for the same purpose in mammals, including long-tailed goral (*Naemorhedus caudatus*) [42] and moose (*Alces alces*) [43]. InDel markers have been used as relatively novel genetic markers in some species such as wild dogs [44] and native chickens and wild fowls in southeast Asia [45], although individual identification was not a major issue in those studies. These InDel mutations were determined by DNA sequencing. As far as we know, however, agarose gel electrophoresis-resolvable InDel markers have only so far been reported in some plant species [24,46]. 

In this study with InDel markers, global Ho and He in a non-relative population of red-crowned cranes in eastern Hokkaido were 0.287 and 0.316, respectively. Using six microsatellite markers, Sun et al. [35] reported that averaged Ho and He in a wintering red-crowned crane population in southeastern China (Yancheng) were 0.654 and 0.768, respectively. Although the genetic markers used were different, notable differences in these heterozygosity indexes are additional evidence for scarce genetic diversity in the island population. Low genetic diversity in the island population has been frequently reported based on haplotype analysis [9,10,27] and microsatellite analysis [17,18]. It has been assumed that there was a high degree of inbreeding or genetic drift as the cause of scarce diversity in the island population [10]. In this study, the average of FIS of the 11 InDel markers as an inbreeding index was 0.095. This suggests that no or only a weak trend of inbreeding occurred in the island population. Taking into consideration the fact that about 20 or 40 red-crowned cranes remained in a restricted area in Kushiro Wetland for a long time [13], it is more likely that a bottleneck effect is the cause of scarce genetic diversity in the Hokkaido population [9,10,11].

Sugimoto et al. [18] compared genetic differentiation in three major areas in eastern Hokkaido (Kushiro, Nemuro and Tokachi) using 12 microsatellite markers. A significant difference in FST was only observed between Nemuro and Tokachi. A similar comparison with our InDel markers showed only a week trend of differentiation between Nemuro and Tokachi based on the FST value (0.033, *p* = 0.05). Additionally, significant differentiation was not found in the whole eastern Hokkaido population. Our PCA analysis also ruled out differentiation in these areas. Thus, it is hardly said that the populations of Nemuro and Tokachi are different at the present time.

Averaged concordance rates of two cranes in the blood-relative population (parent–offspring and full-siblings) were slightly but significantly higher than those in the non-relative population. However, concordance rates of these relative populations were largely overlapping with those of the non-relative population (Figure 2). Nevertheless, if InDel information on couples and a certain crane is available, the parent and child relationship can be estimated, although accuracy is not sufficiently high (93.9%). In normal distribution plots, the lowest limit of the upper 5% population of non-relatives was 89.1%, suggesting that two cranes with a concordance rate of more than 89.1% in InDel patterns can be estimated as parent–offspring. The highest limits of the lower 5% population of parent–offspring and full-siblings were 71.6% and 67.9%, respectively. This indicates that two cranes with a concordance rate of less than 67.9% can be regarded as non-relatives. Thus, the InDel markers that we found can be used for the identification of relatives almost without exception when the concordance of two cranes is very high. Combined information on sex, haplotype and other markers such as microsatellite and cytochrome B type would contribute to a more reliable determination of relatives.

## 5. Conclusions

This is the first report on conventional gel electrophoresis-resolvable InDel markers in animal species, as far as we know. InDel PCR is highly accurate for individual identification compared to the other methods, including the method with microsatellite markers. The method has additional advantages, such as convenience and low cost without sequencing and an expensive apparatus. Since it was suggested that red-crowned cranes in the continental population could invade the island population [11], individual identification may become increasingly important for other behavioral studies as well.

## Figures and Tables

**Table 1:** Eleven InDel markers and primers to detect them.

InDel	Chromosome (Position)	InDel Type	Indel Size (bp)	Forward Primer (5′-3′)	Reverse Primer (5′-3′)	PCR Product (bp)
Id-01	Grja_16152 (2703..2734)	Deletion	32	AATTCAGTGGGTTTGCTCCCGCGGAA	TCAGTTCGTGTCGTACGTTGTCCCA	200
Id-02	Grja_24077 (3699..3730)	Deletion	32	AGCAGCTCTGGAGTCACCAAAGCA	TCAAAATGGTTCCATGTGCCCTGTGT	205
Id-03	Grja_10155 (1061..1090)	Insertion	30	GCAGGATAGCAGCCTAATTGTATCAA	AGGTAGGGCAACTTTAAAGTACTTTGA	160
Id-04	Grja_25718 (20476..20505)	Deletion	30	AAAGGAGGGAATTTGCAGAGCCCA	TGTGTTTTCTGAGACTTTCCACTCT	234
Id-05	Grja_8974 (39698..39727)	Deletion	30	TGGTATGTCTGCCACTCAGAAAGGGA	AGAAACAGGTAAAGGCTCACACCAGA	207
Id-06	Grja_3909 (47326..47361)	Deletion	36	TGACTACCTGTGCACCCAGACAGCA	ATGGTGTGGTGGCATGCCAGCAAATA	147
Id-07	Grja_36880 (1992..2021)	Deletion	30	ATGTCTCTCTTGTGTCCCCTTGGGA	ACAGCCCAGAGATGGTGGCCACAA	175
Id-08	Grja_29257 (18954..18984)	Deletion	31	TAGCAAAGCTCCAGAACAGGACTCCT	AGAGTTTCAGTACAGAAAGACCTGCT	236
Id-9	Grja_13764 (9525..9554)	Deletion	30	TATGGTAACGGAACAGGGAGGGGGT	TGTCCCTTGTTTCAGTTCCAGCAGCT	251
Id-10	Grja_2883 (30876..30905)	Deletion	30	ACATGTTGGCCTTTAAAGGCTGAGCA	AGGAGCACCGACTAGCATTCAGCAT	183
Id-11	Grja_3871 (53311..53340)	Deletion	30	ACCTCACTACTAGCTCCTTATTCA	TTAGATAAGCCTGCCTCTGTAACTA	180

Three percent agarose gel electrophoresis resolvable InDel markers were selected. Chromosome positions in genome information of red-crowned crane (DDDBJ: LC713377) are indicated.

**Table 2:** Summarized InDel patterns and the other information of non-kin individual red-crowned cranes randomly selected from areas of Kushiro, Nemuro and Tokachi.

				InDel Marker										
Area	Crane	Sex	Haplotype	Id-01	Id-02	Id-03	Id-04	Id-05	Id-06	Id-07	Id-08	Id-9	Id-10	Id-11
Kushiro (13)	70	Female	Gj2	W Homo	D Homo	WI Hetero	W Homo	W Homo	WD Hetero	D Homo	W Homo	W Homo	W Homo	W Homo
	89	Female	Gj1	W Homo	W Homo	WI Hetero	W Homo	WD Hetero	WD Hetero	WD Hetero	W Homo	W Homo	WD Hetero	W Homo
	123	Male	Gj1	W Homo	W Homo	W Homo	W Homo	WD Hetero	W Homo	W Homo	W Homo	W Homo	W Homo	W Homo
	150	Male	Gj2	W Homo	WD Hetero	I Homo	W Homo	WD Hetero	W Homo	WD Hetero	WD Hetero	W Homo	WD Hetero	W Homo
	194	Female	Gj1	W Homo	W Homo	W Homo	W Homo	WD Hetero	W Homo	D Homo	W Homo	W Homo	W Homo	W Homo
	211	Male	Gj2	W Homo	W Homo	W Homo	W Homo	W Homo	WD Hetero	W Homo	WD Hetero	W Homo	W Homo	W Homo
	247	Male	Gj2	W Homo	D Homo	W Homo	W Homo	W Homo	W Homo	WD Hetero	WD Hetero	W Homo	WD Hetero	W Homo
	251	Female	Gj2	W Homo	W Homo	W Homo	W Homo	W Homo	W Homo	WD Hetero	W Homo	W Homo	W Homo	W Homo
	271	Male	Gj2	W Homo	W Homo	W Homo	W Homo	W Homo	W Homo	W Homo	WD Hetero	WD Hetero	W Homo	W Homo
	315	Female	Gj2	WD Hetero	WD Hetero	I Homo	WD Hetero	WD Hetero	W Homo	D Homo	W Homo	WD Hetero	W Homo	WD Hetero
	344	Male	Gj2	W Homo	W Homo	WI Hetero	W Homo	W Homo	WD Hetero	W Homo	WD Hetero	WD Hetero	WD Hetero	W Homo
	361	Male	Gj2	W Homo	D Homo	W Homo	W Homo	W Homo	WD Hetero	WD Hetero	W Homo	W Homo	W Homo	W Homo
	362	Male	Gj1	W Homo	W Homo	WI Hetero	W Homo	W Homo	D Homo	W Homo	WD Hetero	W Homo	WD Hetero	W Homo
Nemuro (13)	54	Male	Gj2	W Homo	W Homo	WI Hetero	W Homo	W Homo	WD Hetero	WD Hetero	WD Hetero	W Homo	WD Hetero	W Homo
	72	Female	Gj2	W Homo	W Homo	WI Hetero	W Homo	WD Hetero	W Homo	WD Hetero	W Homo	W Homo	WD Hetero	W Homo
	127	Male	Gj2	W Homo	W Homo	WI Hetero	W Homo	WD Hetero	WD Hetero	WD Hetero	W Homo	W Homo	WD Hetero	W Homo
	137	Male	Gj2	W Homo	W Homo	WI Hetero	W Homo	W Homo	WD Hetero	WD Hetero	WD Hetero	W Homo	D Homo	W Homo
	139	Female	Gj2	W Homo	W Homo	W Homo	W Homo	W Homo	WD Hetero	W Homo	W Homo	W Homo	W Homo	W Homo
	223	Male	Gj2	WD Hetero	WD Hetero	W Homo	WD Hetero	W Homo	WD Hetero	WD Hetero	WD Hetero	W Homo	D Homo	W Homo
	280	Female	Gj2	W Homo	WD Hetero	W Homo	WD Hetero	W Homo	W Homo	W Homo	W Homo	W Homo	WD Hetero	W Homo
	287	Female	Gj2	WD Hetero	W Homo	W Homo	W Homo	WD Hetero	W Homo	WD Hetero	W Homo	W Homo	D Homo	W Homo
	319	Male	Gj2	W Homo	WD Hetero	W Homo	WD Hetero	WD Hetero	W Homo	W Homo	W Homo	W Homo	D Homo	W Homo
	330	Female	Gj2	W Homo	D Homo	I Homo	W Homo	W Homo	WD Hetero	D Homo	D Homo	W Homo	W Homo	W Homo
	331	Female	Gj2	W Homo	WD Hetero	I Homo	W Homo	W Homo	WD Hetero	W Homo	W Homo	W Homo	W Homo	W Homo
	396	Female	Gj2	W Homo	WD Hetero	I Homo	W Homo	WD Hetero	WD Hetero	W Homo	D Homo	W Homo	D Homo	W Homo
	397	Female	Gj2	W Homo	W Homo	W Homo	W Homo	W Homo	D Homo	WD Hetero	W Homo	W Homo	W Homo	W Homo
Tokachi (13)	63	Female	Gj2	W Homo	WD Hetero	W Homo	WD Hetero	W Homo	W Homo	WD Hetero	W Homo	W Homo	WD Hetero	W Homo
	71	Female	Gj2	W Homo	D Homo	I Homo	W Homo	WD Hetero	W Homo	WD Hetero	W Homo	W Homo	W Homo	W Homo
	78	Male	Gj2	W Homo	W Homo	WI Hetero	W Homo	W Homo	W Homo	WD Hetero	W Homo	W Homo	W Homo	D Homo
	109	Female	Gj2	W Homo	WD Hetero	W Homo	W Homo	WD Hetero	W Homo	WD Hetero	W Homo	WD Hetero	WD Hetero	W Homo
	131	Female	Gj2	WD Hetero	WD Hetero	I Homo	W Homo	W Homo	W Homo	W Homo	W Homo	W Homo	W Homo	W Homo
	187	Male	Gj2	W Homo	W Homo	WI Hetero	W Homo	W Homo	W Homo	W Homo	W Homo	W Homo	W Homo	W Homo
	201	Male	Gj2	WD Hetero	D Homo	W Homo	W Homo	W Homo	WD Hetero	W Homo	W Homo	WD Hetero	WD Hetero	W Homo
	207	Male	Gj2	W Homo	WD Hetero	I Homo	W Homo	WD Hetero	WD Hetero	W Homo	W Homo	W Homo	WD Heter	W Homo
	213	Female	Gj2	W Homo	W Homo	W Homo	W Homo	W Homo	W Homo	WD Hetero	W Homo	W Homo	WD Heter	W Homo
	254	Female	Gj2	WD Hetero	WD Hetero	W Homo	W Homo	WD Hetero	W Homo	W Homo	D Homo	WD Hetero	W Homo	WD Hetero
	256	Female	Gj2	W Homo	W Homo	WI Hetero	W Homo	W Homo	W Homo	W Homo	D Homo	W Homo	W Homo	WD Hetero
	371	Male	Gj1	WD Hetero	WD Hetero	W Homo	W Homo	WD Hetero	W Homo	W Homo	W Homo	WD Hetero	WD Heter	W Homo
	373	Female	Gj1	WD Hetero	W Homo	W Homo	W Homo	W Homo	WD Hetero	WD Hetero	W Homo	WD Hetero	WD Heter	W Homo

W, I and D are abbreviations of wild allele, insertion allele and deletion allele, respectively. W Homo, I Homo and D Homo indicate the homogenous type of wild, insertion and deletion, respectively. WI and WD mean heterogeneous type of wild-insertion and wild-deletion, respectively. Blood relatives are not included in these cranes used. White for wild type homo (W Homo), dark green for deletion type homo (D Homo), light green for hetero of wild type and deletion type (WD Hetero), dark blue for insertion type homo (I Homo) and light blue for hetero of wild type and insertion type (WI Hetero) are indicated.

**Table 3:** Some parameters of population genetics in Tokachi, Kushiro and Nemuro in eastern Hokkaido using InDel markers.

	N	Ho	He	Fis	P
Tokachi	13	0.301	0.322	0.069	0.423
Kushiro	13	0.266	0.296	0.108	0.255
Nemuro	13	0.294	0.320	0.085	0.415
Total	39	0.287	0.316	0.095	0.075

Thirty-nine red-crowned cranes (non-kin) were selected from areas of Kushiro, Nemuro and Tokachi in eastern Hokkaido. N: observed number of crane individuals, Ho: observed heterozygosity, He: expected heterozygosity, Fis: the inbreeding coefficient in F-statistics, P: corresponding *p* values in the G-test.

**Table 4:** Pairwise FST values and corresponding *p* values from the G-test for genetic differentiation among groups of Tokachi, Kushiro and Nemuro.

Pop1	Pop2	FST	P
Tokachi	Kushiro	−0.006	0.566
Tokachi	Nemuro	0.033	0.050
Kushiro	Nemuro	0.009	0.289

Thirteen cranes were selected from Kushiro, Nemuro and Tokachi in eastern Hokkaido, respectively. FST: pairwise fixation index. P: corresponding *p* values in the G-test. Global FST was 0.013 (*p* = 0.337) (N = 39).

**Table 5:** Summarized InDel patterns and the other information of individual red-crowned cranes in a population containing blood relatives.

				InDel Marker										
Banding No.	Relationship	Sex	Haplotype	Id-01	Id-02	Id-03	Id-04	Id-05	Id-06	Id-07	Id-08	Id-9	Id-10	Id-11
207	Father	Male	Gj2	W Homo	WD Hetero	I Homo	W Homo	WD Hetero	WD Hetero	W Homo	W Homo	W Homo	WD Hetero	W Homo
352	Sibling of 207	Male	Gj2	W Homo	WD Hetero	I Homo	W Homo	WD Hetero	WD Hetero	W Homo	W Homo	W Homo	W Homo	W Homo
387	Ditto	Female	Gj2	W Homo	W Homo	I Homo	W Homo	WD Hetero	W Homo	W Homo	W Homo	W Homo	WD Hetero	WD Hetero
388	Ditto	Female	Gj2	W Homo	WD Hetero	I Homo	W Homo	W Homo	WD Hetero	W Homo	W Homo	W Homo	W Homo	W Homo
213	Mother	Female	Gj2	W Homo	W Homo	W Homo	W Homo	W Homo	W Homo	WD Hetero	W Homo	W Homo	WD Hetero	W Homo
374	Sibling of 213	Female	Gj2	W Homo	W Homo	W Homo	W Homo	WD Hetero	W Homo	W Homo	W Homo	W Homo	WD Hetero	W Homo
T73/R568	Father of 116-390	Male	Gj2	W Homo	WD Hetero	WI Hetero	W Homo	WD Hetero	W Homo	WD Hetero	WD Hetero	W Homo	W Homo	W Homo
116	Mother of 358, 359	Female	Gj2	WD Hetero	W Homo	W Homo	W Homo	D Homo	W Homo	W Homo	D Homo	W Homo	WD Hetero	WD Hetero
204	Sibling of T73	Female	Gj2	WD Hetero	WD Hetero	I Homo	W Homo	W Homo	W Homo	WD Hetero	W Homo	W Homo	W Homo	WD Hetero
261	Ditto	Female	Gj2	WD Hetero	W Homo	W Homo	W Homo	WD Hetero	W Homo	WD Hetero	D Homo	W Homo	W Homo	W Homo
312	Ditto	Female	Gj2	WD Hetero	W Homo	W Homo	W Homo	WD Hetero	W Homo	WD Hetero	D Homo	W Homo	WD Hetero	WD Hetero
313	Ditto	Female	Gj2	WD Hetero	WD Hetero	W Homo	W Homo	WD Hetero	W Homo	WD Hetero	W Homo	W Homo	W Homo	W Homo
353	Ditto	Male	Gj2	WD Hetero	WD Hetero	I Homo	W Homo	WD Hetero	W Homo	WD Hetero	WD Hetero	W Homo	W Homo	W Homo
390	Ditto	Female	Gj2	WD Hetero	WD Hetero	W Homo	W Homo	D Homo	W Homo	W Homo	D Homo	W Homo	WD Hetero	W Homo
253	Grandsibling of T73	Male	Gj2	WD Hetero	W Homo	WI Hetero	W Homo	WD Hetero	W Homo	D Homo	WD Hetero	W Homo	W Hetero	W Homo
384	Ditto	Female	Gj2	WD Hetero	W Homo	W Homo	W Homo	WD Hetero	W Homo	D Homo	W Homo	W Homo	W Hetero	W Homo
358	Ditto	Male	Gj2	W Homo	W Homo	W Homo	W Homo	WD Hetero	W Homo	W Homo	WD Hetero	W Homo	WD Hetero	WD Hetero
359	Ditto	Female	Gj2	W Homo	W Homo	W Homo	W Homo	WD Hetero	W Homo	W Homo	W Homo	W Homo	WD Hetero	WD Hetero
123	Father	Male	Gj1	W Homo	W Homo	W Homo	W Homo	WD Hetero	W Homo	W Homo	W Homo	W Homo	W Homo	W Homo
131	Mother	Female	Gj2	WD Hetero	WD Hetero	I Homo	W Homo	W Homo	W Homo	W Homo	W Homo	W Homo	W Homo	W Homo
244	Sibling of 123 and 131	Male	Gj2	W Homo	W Homo	WI Hetero	W Homo	WD Hetero	W Homo	W Homo	W Homo	W Homo	W Homo	W Homo
245	Twins of 244	Female	Gj2	W Homo	WD Hetero	W Homo	W Homo	WD Hetero	W Homo	W Homo	W Homo	W Homo	W Homo	W Homo
211	Father	Male	Gj2	W Homo	W Homo	W Homo	W Homo	W Homo	WD Hetero	W Homo	WD Hetero	W Homo	W Homo	W Homo
362	Sibling of 211	Male	Gj1	W Homo	W Homo	WI Hetero	W Homo	W Homo	D Homo	W Homo	WD Hetero	W Homo	WD Hetero	W Homo
315	Twins of 316	Female	Gj2	WD Hetero	WD Hetero	I Homo	WD Hetero	WD Hetero	W Homo	D Homo	W Homo	WD Hetero	W Homo	WD Hetero
316	Twins of 315	Male	Gj2	W Homo	W Homo	WI Hetero	WD Hetero	W Homo	W Homo	WD Hetero	WD Hetero	W Homo	W Homo	WD Hetero
201	Father	Male	Gj2	WD Hetero	D Homo	W Homo	W Homo	W Homo	WD Hetero	W Homo	W Homo	WD Hetero	WD Hetero	W Homo
375	Sibling of 201	Female	Gj2	W Homo	WD Hetero	W Homo	W Homo	W Homo	WD Hetero	W Homo	W Homo	W Homo	WD Hetero	W Homo
63	Mother	Female	Gj2	W Homo	WD Hetero	W Homo	WD Hetero	W Homo	W Homo	WD Hetero	W Homo	W Homo	WD Hetero	W Homo
169	Sibling of 63	Male	Gj2	W Homo	WD Hetero	W Homo	W Homo	W Homo	WD Hetero	WD Hetero	W Homo	WD Hetero	WD Hetero	W Homo
109	Mother	Female	Gj2	W Homo	WD Hetero	W Homo	W Homo	WD Hetero	W Homo	WD Hetero	W Homo	WD Hetero	WD Hetero	W Homo
392	Sibling of 109	Female	Gj2	W Homo	WD Hetero	I Homo	W Homo	D Homo	WD Hetero	WD Hetero	W Homo	W Homo	W Homo	W Homo
T74/R566	Father	Male	-	WD Hetero	WD Hetero	W Homo	W Homo	W Homo	W Homo	W Homo	W Homo	WD Hetero	WD Hetero	W Homo
124	Sibling of T74	Female	Gj1	W Homo	D Homo	W Homo	W Homo	WD Hetero	W Homo	W Homo	W Homo	W Homo	WD Hetero	W Homo
125	Ditto	Male	Gj1	WD Hetero	WD Hetero	W Homo	W Homo	WD Hetero	W Homo	W Homo	W Homo	WD Hetero	W Homo	W Homo
251	Mother	Female	Gj2	W Homo	W Homo	W Homo	W Homo	W Homo	W Homo	WD Hetero	W Homo	W Homo	W Homo	W Homo
415	Sibling of 251	Male	Gj2	W Homo	W Homo	WI Hetero	W Homo	WD Hetero	WD Hetero	WD Hetero	W Homo	WD Hetero	W Homo	W Homo
271	Father	Male	Gj2	W Homo	W Homo	W Homo	W Homo	W Homo	W Homo	W Homo	WD Hetero	WD Hetero	W Homo	W Homo
420	Sibling of 271	Female	Gj1	W Homo	W Homo	W Homo	W Homo	W Homo	W Homo	W Homo	W Homo	WD Hetero	W Homo	W Homo

W, I and D are abbreviations of wild allele, insertion allele and deletion allele, respectively. W Homo, I Homo and D Homo indicate the homogenous type of wild, insertion and deletion, respectively. WI and WD indicate heterogeneous type of wild-insertion and wild-deletion, respectively. White for wild type homo (W Homo), dark green for deletion type homo (D Homo), light green for hetero of wild type and deletion type (WD Hetero), dark blue for insertion type homo (I Homo) and light blue for hetero of wild type and insertion type (WI Hetero) are indicated.

## Data Availability

The datasets generated during and/or analyzed during the current study are available from the corresponding author on reasonable request.

## Supplementary Materials

The following supporting information can be downloaded at: https://www.mdpi.com/article/10.3390/ani12172293/s1, Figure S1: Images of agarose gel electrophoresis of PCR products with 11 InDel primer sets.; Figure S2: Images of agarose gel electrophoresis of PCR products with genomic DNA from feather shafts; Figure S3: Images of agarose gel electrophoresis of PCR products with genomic DNA from intestinal contents and feces; Table S1: Information on red-crowned crane chicks, of which whole blood samples were used for the search of InDel markers; Table S2: Some parameters of population genetics in the eastern Hokkaido population using InDel markers.
