# Supplementary material for: Conventional Gel Electrophoresis-Resolvable Insertion/Deletion Markers for Individual Identification and Analysis of Population Genetics in Red-Crowned Cranes in Eastern Hokkaido, Japan
Source: Animals (Basel). 2022 Sep 4;12(17):2293. doi: 10.3390/ani12172293 (PMC9455020; doi:10.3390/ani12172293)
Supplement: Supplementary file 1 [file animals-12-02293-s001.zip › animals-1880227-supplementary.pdf]

Table S1 Information on red-crowned crane chicks, which whole blood samples were used for search of InDel markers

	Collection location	Collection date	Sex	Haplotype
318	Kiyozumi Beach, Atsuma (42.3542 141.5138)	July 14, 2017	Male	Gj1
380	Koitoi-numa Pond, Shiranuka (42.9996 144.1608)	July 1, 2020	Female	Gj2
389	Ushikubibetsu, Toyokoro (42.7895 143.4555)	July 7, 2020	Male	Gj2
391	Tohoro bridge, Nakashibetsu (43.5284 145.0019)	July 11, 2020	Female	Gj2
394	Satsutomonai, Teshikaga (43.5203 144.4301)	July 11, 2020	Male	Gj2
395	Kitoushi, Ashoro (43.3590 143.4015)	July 12, 2020	Female	Gj1

Figures inside parentheses in collection location indicate the latitude/longitude. All blood samples were collected from wild chicks in Hokkaido, Japan.

Table S2 Some parameters of population genetics in eastern Hokkaido population using InDel markers

	InDel Markers											
	Id-01	Id-02	Id-03	Id-04	Id-05	Id-06	Id-07	Id-08	Id-09	Id-10	Id-11	Average
K	2	2	2	2	2	2	2	2	2	2	2	2
N	39	39	39	39	39	39	39	39	39	39	39	39
Ho	0.205	0.333	0.308	0.128	0.385	0.41	0.462	0.231	0.205	0.41	0.077	0.2867
He	0.186	0.441	0.475	0.122	0.315	0.386	0.45	0.345	0.186	0.45	0.122	0.3162
Fis	-0.1294	0.2642	0.3739	-0.0526	-0.2000	-0.1098	-0.0286	0.3494	-0.1566	0.0154	0.3455	0.06104
PIC	0.167	0.341	0.37	0.113	0.262	0.309	0.346	0.283	0.167	0.346	0.113	0.2561
NEP	0.683	0.413	0.381	0.782	0.523	0.455	0.407	0.493	0.683	0.407	0.782	0.5463
P	1.0000	0.1514	0.0311	1.0000	0.3073	1.0000	1.0000	0.0531	1.0000	0.7216	0.1275	0.5811

Thirty-nine red-crowned cranes that did not contain blood relatives were selected from areas of Kushiro, Nemuro and Tokachi in eastern Hokkaido. K: number of alleles, N: observed number of crane individuals, Ho: observed heterozygosity, He: expected heterozygosity, PIC：polymorphic information content, NEP: non-exclusion probabilities, P: corresponding *p* values in the G-test.

Figure S1 Images of agarose gel electrophoresis of PCR products with 11 InDel primer sets.

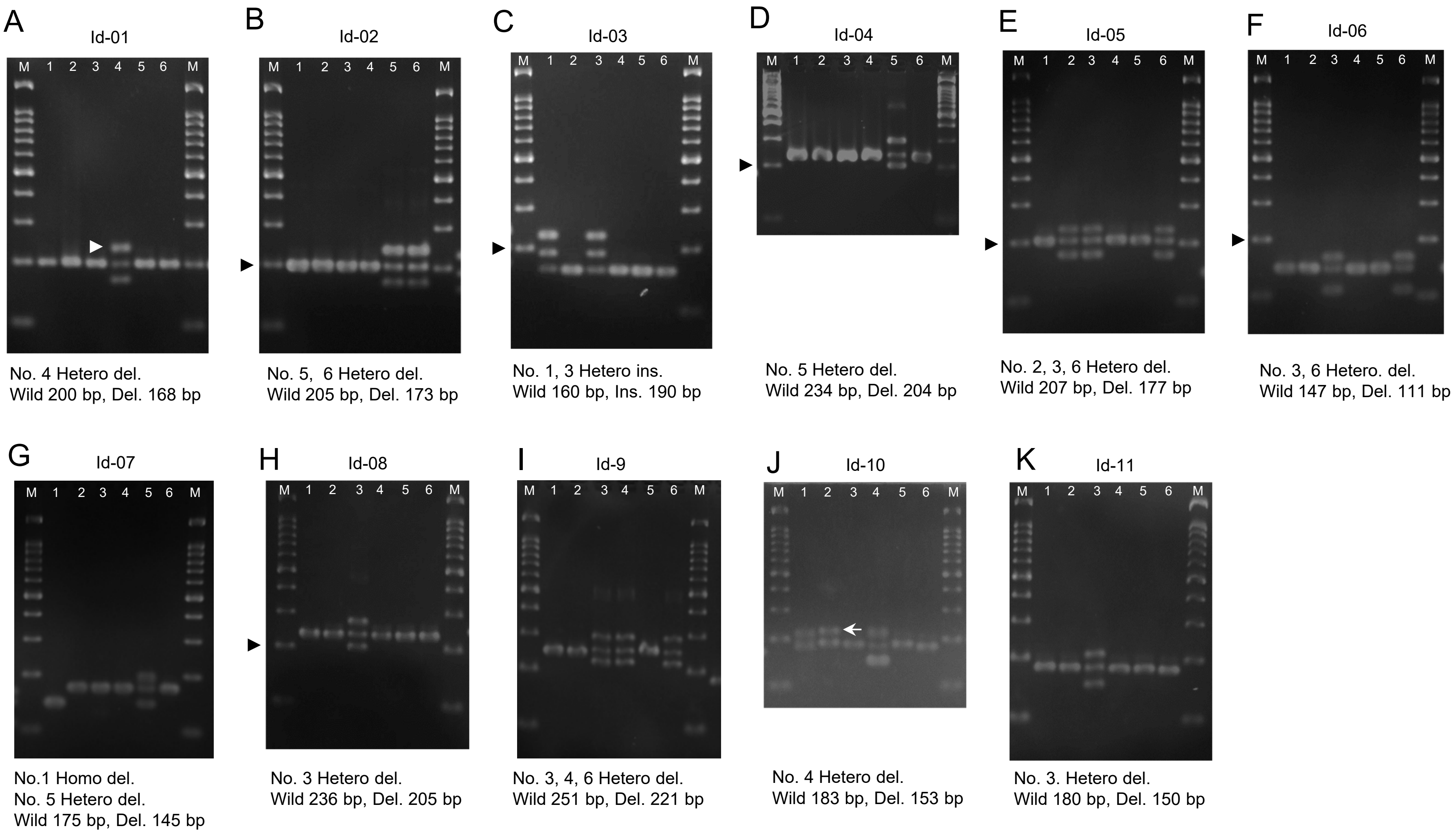

Figure S1. Blood samples used were chick No. 318 for lane 1, No. 380 for lane 2, No. 389 for lane 3, No. 391 for lane 4, No. 394 for lane 5 and No. 395 for lane 6 (Table S1). M in each gel image indicates a 100-bp ladder size marker and black arrowheads indicate 200 bp. "Del. (del.)" and "Ins. (ins.)" are abbreviations of deletion allele and insertion allle, respectively. "Wild" means wild-type allele. The white arrowhead in panel A indicates the band of a heterodimer of PCR products for an example. White arrows in lane 1 and 2 of panel J indicate unidentified bands.

Figure S2 Images of agarose gel electrophoresis of PCR products with genomic DNA from feather shafts.

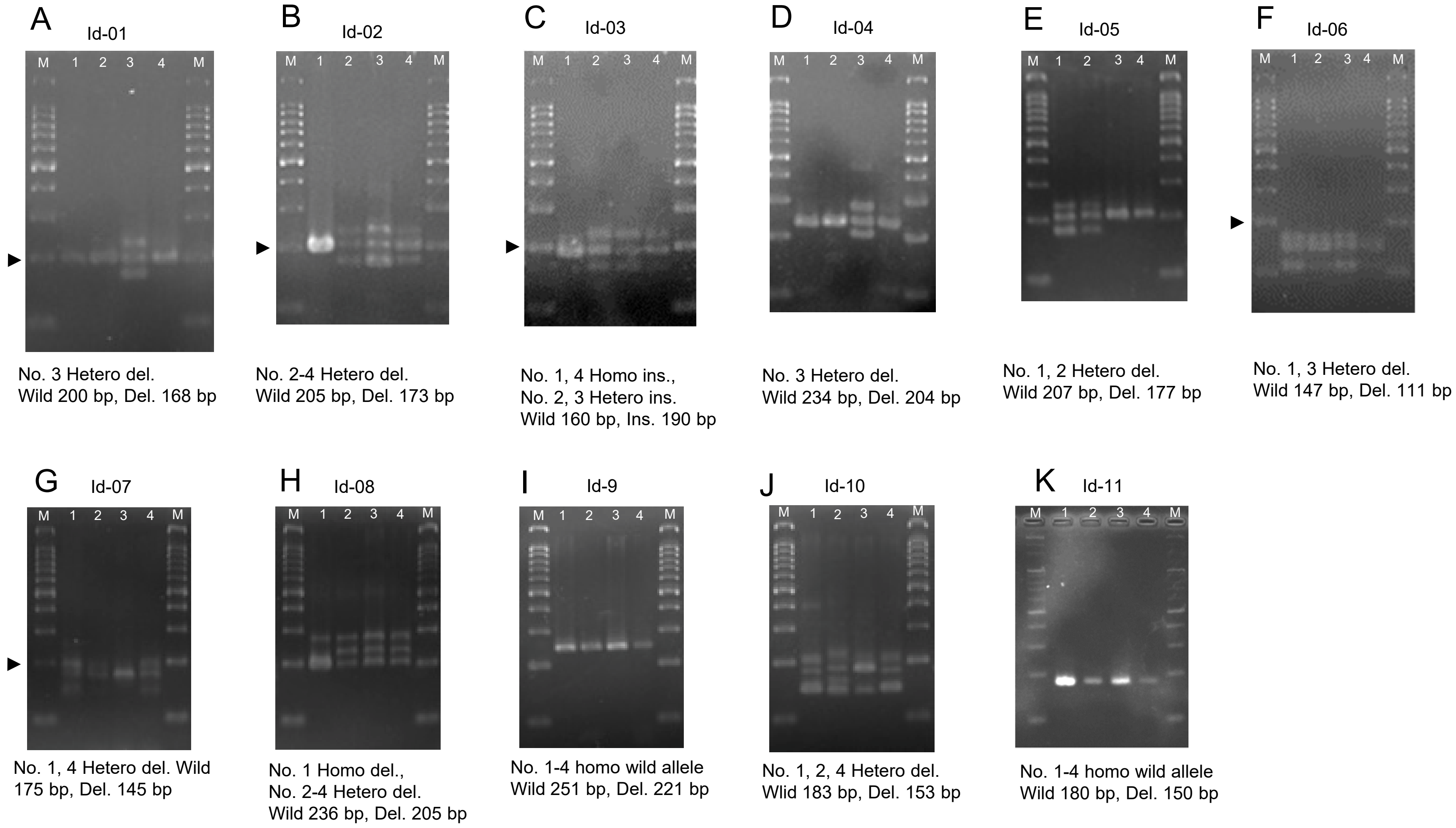

Figure. S2. Images of agarose-gel electrophoresis of PCR products with 11 InDel primer sets. Samples used were R310 for lane 1, R353 for lane 2, R371 for lane 3, R380 for lane 4. M in each gel image indicates 100 bp ladder size marker and black arrowheads indicate 200 bp. Del. and Ins. are abbreviations of deletion and insertion, respectively.

Figure. S3 Images of agarose gel electrophoresis of PCR products with genomic DNA from intestinal contents and feces.

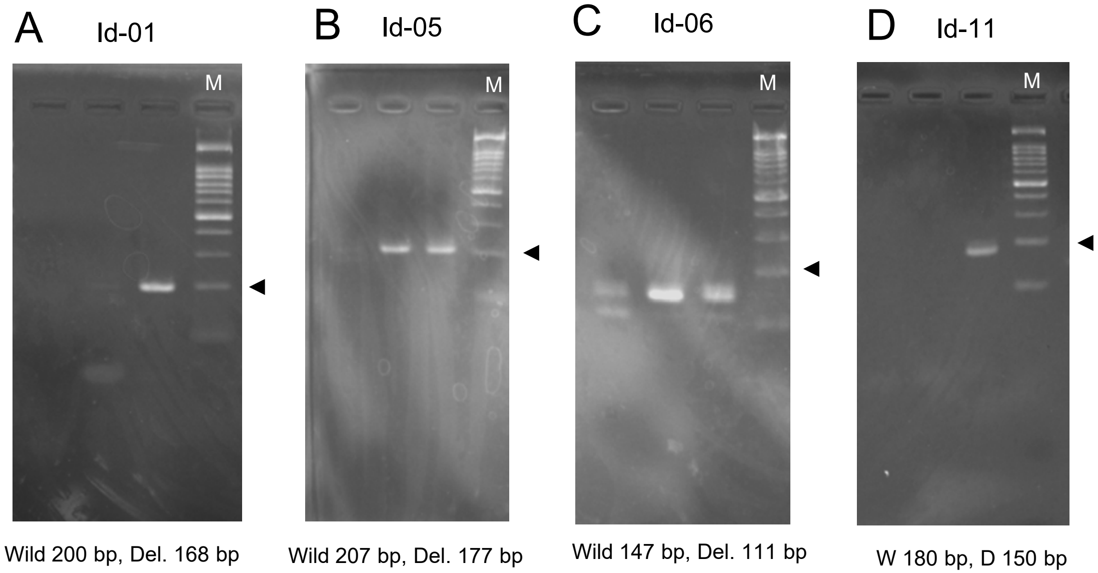

Images of agarose-gel electrophoresis of PCR products with primer sets Id-01, Id-05, Id-06 and Id-11 among 11 InDel primer sets. Samples used were R561 for lane 1, R566 for lane 2 and 316 for lane 3. R561 and R566 are DNA extracts from intestinal contents. 316 is DNA extract from feces of 316. M in each gel image indicates 100 bp ladder size marker and black arrowheads indicate 200 bp. Del. is an abbreviation of deletion.
